# Economics of chronic diseases protocol: cost-effectiveness modelling and the future burden of non-communicable disease in Europe

**DOI:** 10.1186/1471-2458-14-456

**Published:** 2014-05-16

**Authors:** Diana Divajeva, Tim Marsh, Susanne Logstrup, Marleen Kestens, Pepijn Vemer, Vilma Kriaucioniene, Sophie Peresson, Sophie O’Kelly, Ana Rito, Laura Webber

**Affiliations:** 1 UK Health Forum, Fleetbank House, 2-6 Salisbury Square, EC4Y 8JX London, UK; 2 European Heart Network, Rue Montoyer 31, 1000 Brussels, Belgium; 3 PharmacoEpidemiology & PharmacoEconomics (PE2), University of Groningen, Groningen, the Netherlands; 4 Department of Epidemiology, University Medical Centre Groningen, Groningen, the Netherlands; 5 Lithuanian University of Health Sciences, A. Mickevičiaus g. 9, LT 44307 Kaunas, Lithuania; 6 International Diabetes Federation European region (IDF Europe), 166 Chaussee de La Hulpe, B-1170 Brussels, Belgium; 7 European Society of Cardiology, 2035 Route des Colles - Les Templiers, 06903 Sophia Antipolis, France; 8 National Institute of Health Doutor Ricardo Jorge, I.P. Av. Padre Cruz, 1649-016 Lisbon, Portugal

**Keywords:** Chronic disease, Cost-effectiveness, Europe, Modelling, Future burden

## Abstract

**Background:**

The majority of chronic disease is caused by risk factors which are mostly preventable. Effective interventions to reduce these risks are known and proven to be applicable to a variety of settings. Chronic disease is generally developed long before the fatal outcome, meaning that a lot of people spend a number of years in poor health. Effective prevention measures can prolong lives of individuals and significantly improve their quality of life. However, the methods to measure cost-effectiveness are a subject to much debate. The *Economics of Chronic Diseases* project aims to establish the best possible methods of measuring cost-effectiveness as well as develop micro-simulation models apt at projecting future burden of chronic diseases, their costs and potential savings after implementation of cost-effective interventions.

**Method:**

This research project will involve eight European countries: Bulgaria, Finland, Greece, Lithuania, The Netherlands, Poland, Portugal and the United Kingdom (UK). A literature review will be conducted to identify scientific articles which critically review the methods of cost-effectiveness. Contact will be made health economists to inform and enrich this review. This evidence will be used as a springboard for discussion at a meeting with key European stakeholders and experts with the aim of reaching a consensus on recommendations for cost-effectiveness methodology. Epidemiological data for coronary heart disease, chronic kidney disease, type 2 diabetes and chronic obstructive pulmonary disease will be collected along with data on time trends in three major risk factors related to these diseases, specifically tobacco consumption, blood pressure and body mass index. Economic and epidemiological micro-simulation models will be developed to asses the future distributions of risks, disease outcomes, healthcare costs and the cost-effectiveness of interventions to reduce the burden of chronic diseases in Europe.

**Discussion:**

This work will help to establish the best methods of measuring cost-effectiveness of health interventions as well as test a variety of scenarios to reduce the risk factors associated with selected chronic diseases. The modelling projections could be used to inform decisions and policies that will implement the best course of action to curb the rising incidence of chronic diseases.

## Background

The last millennium was characterised by a worldwide epidemiological transition, whereby chronic diseases overtook once predominant infectious diseases as the leading cause of morbidity and mortality [1]. In Europe 87% of all deaths occur due to chronic diseases [2], and the number of people affected by these diseases is expected to rise substantially over the next few decades [3]. Common and preventable risk factors such as hypertension, tobacco use, raised blood glucose, and overweight and obesity are the leading causes of major chronic diseases such as type 2 diabetes, cardiovascular and respiratory diseases and should therefore be targeted if the upward trend in chronic disease morbidity and mortality is to be abated [4].

Rapid increases in chronic diseases impose large human, social and economic costs, putting additional pressure on already overstretched health and social care systems. As a result, searching for cost-effective measures to reduce the burden of chronic diseases has become an important task in public health policy. Moreover, the present climate of austerity across Europe has increased the urgency with which we must find cost-effective solutions. Population-wide interventions in combination with interventions targeted to high-risk individuals and groups, may produce significant shifts in risk factors, which in turn may greatly reduce the burden of chronic diseases in populations [5].

The World Health Organization (WHO) has established a set of “best buys” for interventions that are known to be effective, feasible and affordable in reducing the major risk factors of chronic diseases [5]. On a population level such interventions include ban on smoking in public places, increasing taxes on tobacco and alcohol, and raising public awareness on benefits of a healthy diet and physical activity [5]. In addition to population-wide approaches, screening and pharmacological treatment of individuals who are at high risk of a chronic disease can considerably improve the outcomes, especially when combined with lifestyle interventions. For example, a regimen of aspirin, statins and blood pressure lowering agents can significantly reduce the risk of vascular events in people at high risk of cardiovascular disease and, alongside smoking cessation, is considered a “best buy” [5].

The WHO endorsement of several types of interventions is generally acknowledged as good practice even if methodological challenges in regards to the methods of measuring the cost-effectiveness of interventions remain. Firstly, different guidelines and cut-off levels of what is considered cost-effective as well as differences in discounting rates exist across different countries making it difficult to compare the cost-effectiveness of identical or similar interventions. Secondly, the cost-effectiveness of the same interventions varies considerably according to regional or national contexts and population characteristics [6]. Thirdly, research and policy processes may be hindered by the scarcity of economic and epidemiological data required for cost-effectiveness analysis. These challenges reduce the transferability of interventions and the ability to compare their effectiveness across different settings.

Good health benefits the whole of the society and is essential for economic and social development [7]. However, health is unevenly distributed both between and within countries. Evidence shows that in many countries the gap in health outcomes between the highest and the lowest social strata has increased in recent years [8]. In Europe, these inequalities are likely to increase further as the health effects of the 2008 economic crisis have started to emerge in several countries [9]. People in lower education, employment and/or income systematically experience higher chronic disease morbidity and mortality levels [10]. Measures to halt and reverse rising trends in chronic diseases could and must play an important role in reducing health inequalities.

The *Economics of Chronic Diseases* (EConDA) project addresses the issues outlined above by examining the cost-effectiveness of interventions to prevent, screen and treat four major chronic diseases: coronary heart disease (CHD), type 2 diabetes, chronic kidney disease (CKD) and chronic obstructive pulmonary disease (COPD). The project has two key aims. The first aim is to aid EU Member States in reviewing methods used assessing the cost-effectiveness of interventions and establishing agreement on general principles for good practice in cost-effectiveness analysis based on effective chronic disease interventions. Following the agreement, the second aim is to develop economic and epidemiological demonstration models to address cost-effectiveness of various interventions for chronic disease prevention, as well as to demonstrate differential effects of interventions on various population sub-groups.

The models will examine the effect of interventions on the occurrence of selected chronic diseases and their outcomes. In particular, they will assess the effect of cost-effective interventions on the prevalence of risk factors such as hypertension, body mass index (BMI) and smoking and the resulting change in incidence of and mortality from the four selected diseases.

## Methods

Eight associated partner organisations and ten collaborating partner organisations are involved in the project and eight European countries (Bulgaria, Finland, Greece, Lithuania, the Netherlands, Poland, Portugal and the UK) will be studied. The range of countries was chosen to provide a geographical spread and to examine the impacts of economic and health systems differences in feasibility and applicability of interventions. The work is planned to be concluded in two and a half years, starting in April 2013 and ending September 2015.

EConDA comprises of seven work packages:

1) Coordination

2) Dissemination

3) Evaluation

4) Building a consensus

5) Development of a disease model

6) Development of a cost-effectiveness model

7) Validation of the models

Work on each of the packages will be split between the eight associated partners with one of the partners leading on a selected package. The first three will incorporate administrative matters, including regular meetings, accountability and dissemination of project development stages and results. Methods of the four remaining work packages (4–7) are discussed in more detail below.

### Consensus building – work package 4

Work package 4 will be implemented in three phases. Firstly, a literature review will be conducted to identify the studies which critically assess the methods used to measure the cost-effectiveness of interventions for the selected diseases. The results of the review will be used in shaping the questions of the interviews with leading health economists.

In the second phase, international health economics experts will be consulted in order to enrich the review and gain a deeper understanding of the methods of measuring cost-effectiveness of chronic disease interventions.

In the third phase, a consensus meeting will be held, bringing together a group of European experts including health economists, policy makers and experts from the European Chronic Disease Alliance. This meeting will discuss the themes emerging from the first and the second phases of this work package and will aim to establish consensus on which method/s provide the greatest utility, as well as to address the question of how we measure the cost-effectiveness and the impact of integrated approaches to chronic disease prevention on economies.

Consultation with experts and the consensus meeting will be conducted in line with the European Commission guidelines for European Commission funded projects which are underpinned by the EU Charter of the Fundamental Rights [11]. Since the experts will be consulted in their professional capacity as stakeholders in this EU project the consultations and the consensus meeting are exempt from ethical approval.

### Development of a disease model – work package 5

Work package 5 will include two phases. Firstly, disease and risk data will be collected by reviewing open access papers in PubMed and Science Direct databases, supplemented by Google Scholar search. If necessary additional data will be collected by searching statistical data bases and reports as well as contacting disease registries and individual researchers in the field.

For the purpose of this project, epidemiological data need to be stratified by age groups and sex, socio-economic groups and where possible should be nationally representative. We will collect the most recent incidence, prevalence, mortality, and survival data. In addition, if available, we will collect country-specific remission and recurrence data for the selected diseases.

Risk data will be collected in a similar manner. BMI prevalence data will be collected if, in addition to the inclusion criteria above, they are categorised according to the WHO definition: normal weight (<25 kg/m^2^), pre-obese (25–29.9 kg/m^2^), and obese (≥30 kg/m^2^). Hypertension data will be included if they are defined according to WHO criteria of 140 mmHg systolic and/or 90 mmHg diastolic blood pressure. In addition to age and sex specific incidence and prevalence, data on hypertension stages will be included if available. Smoking patterns will be assessed by collecting data on frequency, the length of smoking in years and exposure to second hand smoke.

Furthermore, we will identify other possible risk factors for each of the four diseases. The literature assessing the significance of each risk factor will be examined to determine the inclusion of a risk factor into the model. The data on relative risks will be taken from clinical trials and meta-analyses and assumed to have worldwide applicability. Same data collection procedures will apply to all participating countries.

Secondly, the project will develop a demonstration micro-simulation model. The model will apply the methods developed by the National Heart Forum for the English Government Tackling Obesities enquiry [12,13]. This model has been subsequently applied to other risk factors and diseases and validated in over 70 countries both nationally and locally including the US, Russia, Mexico and Brazil. In short, a dual-module Markov-style micro-simulation model will be used. Module 1 uses multi-variate non-linear regression to project risk trends to 2030. Module 2 utilises a micro-simulation model to simulate a virtual population and estimate the future incidence, prevalence and mortality of the diseases of interest.

We will project the trends of hypertension, obesity and tobacco use to 2030 and estimate the number of selected diseases’ cases related to these risk factors. We will then test the impact of effective risk interventions on the future incidence and mortality using several risk reduction scenarios. As an example, previously tested scenarios included testing the effect of mean population BMI reduction by 1% and 5% on the occurrence of obesity-related chronic diseases. Similarly, we would be able to test the effects of reductions in population levels of hypertension and the effects of increase in smoking cessation rates.

### Development of a cost-effectiveness model – work package 6

Work package 6 will be implemented in three phases. Firstly, the data on both direct and indirect costs for each selected disease will be collected for each of the eight countries. The process of data collection will be the same as the disease data collection procedure. Secondly, the intervention scenarios will be developed using information collected in work package 4 and the suggestions arising from the further discussion with partners and experts. Thirdly, following phases one and two an economic model will be developed. A demonstration model will enable to test the cost-effectiveness of interventions to prevent, screen and treat CHD, COPD, type 2 diabetes and CKD.

### Validation of the models – work package 7

We will validate the models using the best available data from one of the eight countries involved in the project. The model will be able to demonstrate the future health impact of positive and negative changes in risk factors as well as indicate the most cost-effective interventions to reduce the burden of chronic disease. Moreover, the model will allow for any specified risk reduction or intervention to be modelled and it could be subsequently updated as new data for current or other countries become available.

This study has been approved by the European Commission Health Programme and the Executive Agency for Health and Consumers.

## Discussion

Rising incidence of chronic diseases poses a great challenge to health systems across the globe. In Europe, chronic diseases are the major cause of mortality and morbidity and the prevalence of chronic diseases is predicted to continue to rise to 2030 as a result of ageing populations and an increase in risk factors such as obesity [6]. There are differences in health outcomes both between and within countries, with people in lower socio-economic position experiencing higher rates of chronic diseases [8]. Health inequalities are likely to increase as a result of European financial crisis, which has put a significant strain on health systems in several member states.

Inequalities-related losses to health annually account for 20% of the costs of health care systems in the European Union as a whole, and as long as health inequalities persist, these losses will continue to accumulate over the years [10]. Moreover, it has been estimated that at least 80% of all heart disease, stroke and type 2 diabetes and at least one third of cancer cases are preventable [7]. This suggests that a significant amount of chronic disease-related costs could be saved by tackling health inequalities and by applying preventative measures proven to be cost-effective. Therefore no government should ignore an opportunity of introducing effective health interventions.

This research will make a significant contribution to new knowledge with respect to reduction of risk factors for chronic diseases and implementation of public health programmes. Specifically, the EConDA project will contribute to public health practice in the following ways:

Firstly, it will address issues in measuring the cost-effectiveness of health interventions and help to establish consensus on which method/s provide the greatest utility. This knowledge could be transferred to different settings and contexts and may aid future decision making.

Secondly, data availability is one of the limitations mentioned in many research publications. We will work closely with the associate partners to collect and synthesise the most recent disease and risk factor data as well as best available economic data across eight European countries. Data sources will be listed in an official report and the outputs of this research will be made available through scientific publications and a dedicated website allowing our findings to be employed in further scholarly and research work.

Thirdly, we will assess the burden of chronic disease in various population groups, including those shown to be most at risk. The effectiveness and the cost-effectiveness of public health interventions to reduce the risk of chronic diseases will be tested for the different strata of populations helping to establish the most effective ways in intervening across different population groups.

Finally, the developed models will enable to test and compare a range of scenarios to tackle chronic disease and can be updated and applied to various settings. The dynamics between BMI, smoking, hypertension and related health outcomes is complex, making it difficult to choose appropriate policy responses. Establishing the most effective actions against the chronic disease epidemic and indicating cost-effective interventions are paramount in order to address the financial strain and capacity issues across European health systems. Our models may be a tool to achieve that.

### Limitations of the study

The models present the best possible attempt to test specific intervention scenarios and to assess the health outcomes of disease interventions, given the presently available data. As better data become available in the future it will be possible to improve the models even further and to better capture the disease processes and the related costs, allowing for a better understanding of how interventions would affect different population groups increasing the validity of the results. The current modelling project will help to highlight the existing data gaps and will identify where governments can gain greater utility of the models by collecting better surveillance data.

## Competing interests

The author declares that they have no competing interests.

## Authors’ contributions

DD prepared the manuscript. TM and LW conceived of the study, participated in its design and coordination and reviewed the manuscript. All authors read and approved the final manuscript. For more information, please visit: http://econdaproject.eu.

## Pre-publication history

The pre-publication history for this paper can be accessed here:

http://www.biomedcentral.com/1471-2458/14/456/prepub
